# Supplementary material for: 4-Octyl itaconate inhibits aerobic glycolysis by targeting GAPDH to exert anti-inflammatory effects
Source: Nat Commun. 2019 Nov 8;10:5091. doi: 10.1038/s41467-019-13078-5 (PMC6841710; doi:10.1038/s41467-019-13078-5)
Supplement: Supplementary file 1 — Supplementary Information [file 41467_2019_13078_MOESM1_ESM.pdf]

# Supplementary Information

# 4-Octyl itaconate inhibits aerobic glycolysis by targeting GAPDH to exert anti-inflammatory effects

Liao et al.

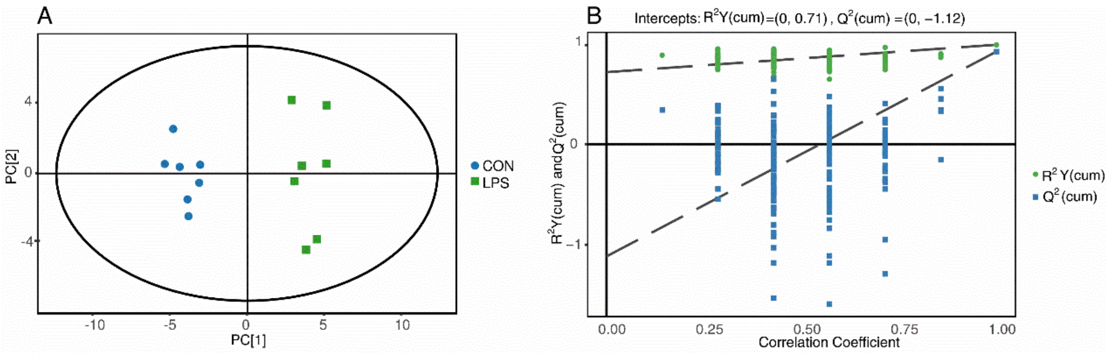


Supplementary Fig. 1 LC-MS/MS metabolomics of control (CON) and LPS-stimulated (LPS) groups. (A), Score plots of the principal component analysis (PCA) model differentiated metabolites of control (CON) and LPS-stimulated (LPS) RAW264.7 macrophages. (B), Scatter plots of the statistical validations obtained by 200 permutation tests for OPLS-DA analysis in RAW264.7 macrophages. Results are from seven independent experiments.

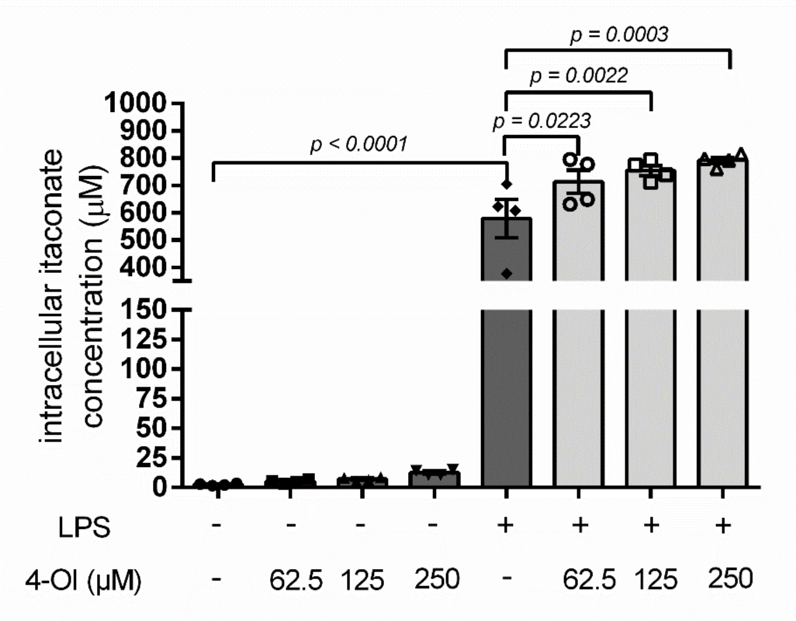


Supplementary Fig. 2 4-OI was hydrolysed to itaconate in LPS stimulated RAW264.7 macrophages. Macrophages were treated with vehicle or 4-OI at the indicated concentrations. After 3 h, cells were stimulated with or without 1 μg/mL LPS for 24 h. Itaconate levels in RAW264.7 macrophages were quantitative by LC-MS/MS. Data represent the mean ± SEM of four independent experiments. *p* values were determined by one-way ANOVA with Sidak's correction for multiple comparisons. Source data are provided as a Source Data file.

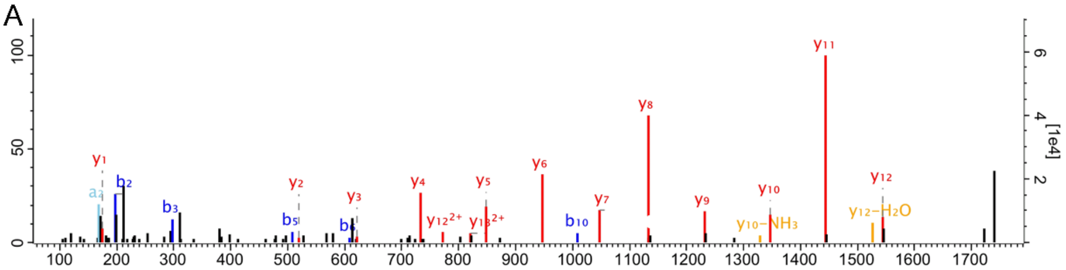


B

| | *b* ion | sequence | *y* ion | |
|---|---|---|---|---|
| 1 | 100.07570 | V | | 14 |
| 2 | 197.12847 | P | 1642.87210 | 13 |
| 3 | 298.17615 | T | 1545.81933 | 12 |
| 4 | 395.22892 | P | 1444.77165 | 11 |
| 5 | 509.27185 | N | 1347.71888 | 10 |
| 6 | 608.34027 | V | 1233.67595 | 9 |
| 7 | 695.37230 | S | 1134.60753 | 8 |
| 8 | 794.44072 | V | 1047.57550 | 7 |
| 9 | 893.50914 | V | 948.50708 | 6 |
| 10 | 1008.53609 | D | 849.43866 | 5 |
| 11 | 1121.62016 | L | 734.41171 | 4 |
| 12 | 1222.66784 | T | 621.32764 | 3 |
| 13 | 1567.82884 | C(+242.15) | 520.27996 | 2 |
| 14 | | R | 175.11896 | 1 |

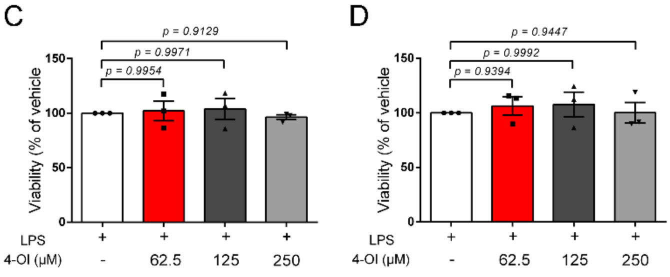


Supplementary Fig. 3 4-OI modified recombinant mouse GAPDH. (A) and (B), Representative LC-MS/MS spectra demonstrated modification of GAPDH cysteine 245 (+242.15 Da) in recombinant mouse GAPDH incubated with 4-OI (500 μM) for 4 h at 37 °C. Da = daltons. (C) and (D), RAW264.7 macrophages (C) or BMDMs (D) were treated with 100 ng/ml or 1 μg/ml LPS plus the indicated doses of 4-OI for 24 h. Viability was assessed by cell counting kit-8. Data represent the mean ± SEM of three experiments performed in duplicate. The results are non-significant by one-way ANOVA with Sidak's correction for multiple comparisons. Source data are provided as a Source Data file.

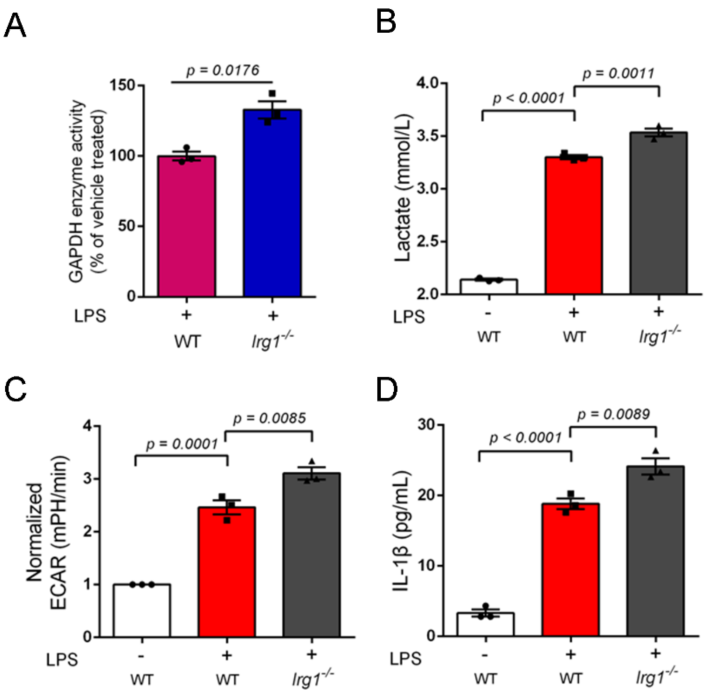


Supplementary Fig. 4 *Irg1* deficiency leads to enhanced GAPDH activity and glycolysis. WT BMDMs and *Irg1*$^{-/-}$ BMDMs were detected for GAPDH enzyme activity (A) after 100 ng/mL LPS stimulation for 24 h. Levels of lactate (B), ECAR (C) and IL-1β (D) were determined in WT and *Irg1*$^{-/-}$ BMDMs with or without LPS stimulation for 24 h. All data shown are summarized from three independent experiments. Values represent the mean ± SEM at each time point. *p* values were calculated using two-tailed Student's t-test or one-way ANOVA with Sidak's correction for multiple comparisons. Source data are provided as a Source Data file.

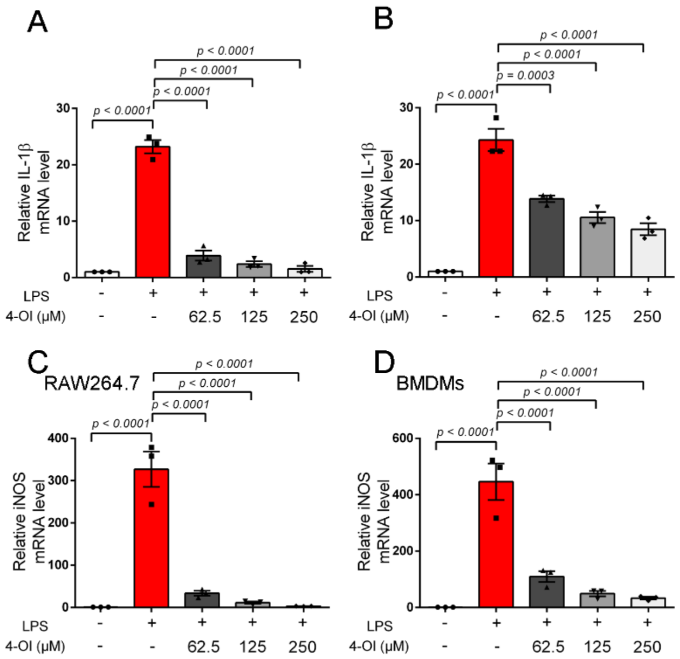


Supplementary Fig. 5 4-OI inhibited IL-1β and iNOS mRNA expressions. Macrophages were treated with vehicle or 4-OI at the indicated concentrations. After 3 h, cells were stimulated with LPS (1 μg/mL or 100 ng/mL) for 24 h. (A) and (B), The mRNA levels of IL-1β in RAW264.7 macrophages (A) and BMDMs (B) were measured by qRT-PCR. (C) and (D), The mRNA expression of IL-1β in RAW264.7 macrophages (C) and BMDMs (D) was identified by qRT-PCR. Data represent the mean ± SEM of three independent experiments. *p* values were determined by one-way ANOVA with Sidak's correction for multiple comparisons. Source data are provided as a Source Data file.

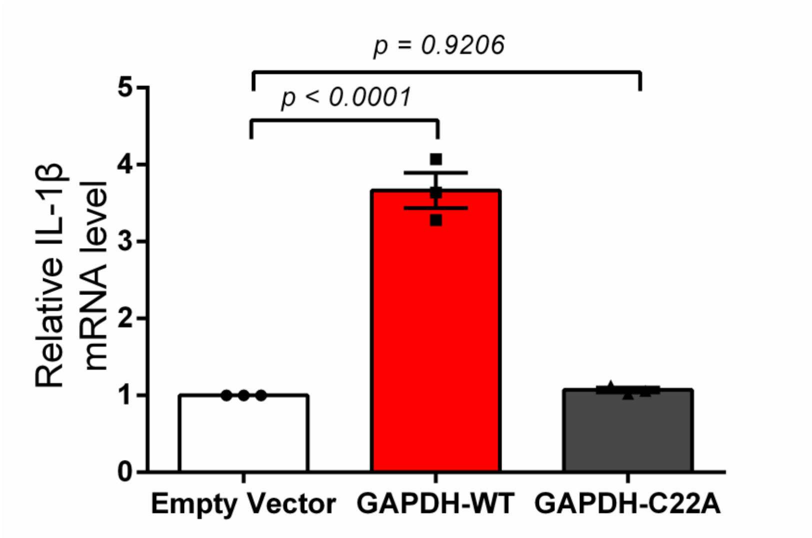


Supplementary Fig. 6 Wild-type GAPDH (GAPDH-WT) abolished the inhibitory effect of 4-OI. GAPDH-WT or the Cys-22 mutant (GAPDH-C22A) was overexpressed in RAW264.7 macrophages for 24 h, and the cells were then treated with LPS and 4-OI for 24 h, followed by measurement of IL-1β mRNA levels by qRT-PCR. Data represent the mean ± SEM of three experiments. *p* values were calculated by one-way ANOVA with Sidak's correction for multiple comparisons. Source data are provided as a Source Data file.

Supplementary Table 1. The sequence of mouse GAPDH peptide

| | *b* ion | sequence | *y* ion | |
|---|---|---|---|---|
| 1 | 72.04440 | A | | 7 |
| 2 | 143.08152 | A | 820.44850 | 6 |
| 3 | 256.16559 | I | 749.41138 | 5 |
| 4 | 601.32659 | C+(242.15) | 636.32731 | 4 |
| 5 | 688.35862 | S | 291.16631 | 3 |
| 6 | 745.38009 | G | 204.13428 | 2 |
| 7 | | K | 147.11281 | 1 |

4-OI modified Cys 22 of GAPDH in RAW264.7 macrophages.

Supplementary Table 2. The sequence of mouse GAPDH peptide

| | *b* ion | sequence | *y* ion | |
|---|---|---|---|---|
| 1 | 72.04440 | A | | 7 |
| 2 | 143.08152 | A | 708.32330 | 6 |
| 3 | 256.16559 | I | 637.28618 | 5 |
| 4 | 489.20139 | C+(130.02) | 524.20211 | 4 |
| 5 | 576.23342 | S | 291.16631 | 3 |
| 6 | 633.25489 | G | 204.13428 | 2 |
| 7 | | K | 147.11281 | 1 |

Itaconate covalently modified Cys 22-containing GAPDH peptide in LPS-induced RAW264.7 macrophages.

Supplementary Table 3. The sequence of recombinant mouse GAPDH peptide

| | *b* ion | sequence | *y* ion | |
|---|---|---|---|---|
| 1 | 72.04440 | A | | 7 |
| 2 | 143.08152 | A | 820.44850 | 6 |
| 3 | 256.16559 | I | 749.41138 | 5 |
| 4 | 601.32659 | C+(242.15) | 636.32731 | 4 |
| 5 | 688.35862 | S | 291.16631 | 3 |
| 6 | 745.38009 | G | 204.13428 | 2 |
| 7 | | K | 147.11281 | 1 |

4-OI covalently modified Cys 22 of recombinant mouse GAPDH.
