# Supplementary material for: 4-Octyl itaconate inhibits aerobic glycolysis by targeting GAPDH to exert anti-inflammatory effects
Source: Nat Commun. 2019 Nov 8;10:5091. doi: 10.1038/s41467-019-13078-5 (PMC6841710; doi:10.1038/s41467-019-13078-5)
Supplement: Supplementary file 3 — Reporting Summary [file 41467_2019_13078_MOESM3_ESM.pdf]

nature research

Corresponding author(s): DBPR NCOMMS-19-15797A

Last updated by author(s): Oct 6, 2019

Reporting Summary

Nature Research wishes to improve the reproducibility of the work that we publish. This form provides structure for consistency and transparency in reporting. For further information on Nature Research policies, see Authors & Referees and the Editorial Policy Checklist .

Statistics

For all statistical analyses, confirm that the following items are present in the figure legend, table legend, main text, or Methods section.

n/a	Confirmed	
☐	☒	The exact sample size (n) for each experimental group/condition, given as a discrete number and unit of measurement
☐	☒	A statement on whether measurements were taken from distinct samples or whether the same sample was measured repeatedly
☐	☒	The statistical test(s) used AND whether they are one- or two-sided<br>*Only common tests should be described solely by name; describe more complex techniques in the Methods section.*
☐	☒	A description of all covariates tested
☐	☒	A description of any assumptions or corrections, such as tests of normality and adjustment for multiple comparisons
☐	☒	A full description of the statistical parameters including central tendency (e.g. means) or other basic estimates (e.g. regression coefficient) AND variation (e.g. standard deviation) or associated estimates of uncertainty (e.g. confidence intervals)
☐	☒	For null hypothesis testing, the test statistic (e.g. F, t, r) with confidence intervals, effect sizes, degrees of freedom and P value noted<br>*Give P values as exact values whenever suitable.*
☒	☐	For Bayesian analysis, information on the choice of priors and Markov chain Monte Carlo settings
☒	☐	For hierarchical and complex designs, identification of the appropriate level for tests and full reporting of outcomes
☒	☐	Estimates of effect sizes (e.g. Cohen's d, Pearson's r), indicating how they were calculated

Our web collection on statistics for biologists contains articles on many of the points above.

Software and code

Policy information about availability of computer code

Data collection	No software was used.
Data analysis	Metabolomic analysis were analysed using R package XCMS (version 3.2) and SIMCA14.1 software package (V14.1, Sartorius Stedim Data Analytics AB, Umea, Sweden). Image Lab software was used for Western blot. Extracellular acidification rate (ECAR) and oxygen consumption rate (OCR) were analysed by Wave (Agilent Technologies, Inc.). GraphPad Prism 6 was used for all graphing and statistical tests.

For manuscripts utilizing custom algorithms or software that are central to the research but not yet described in published literature, software must be made available to editors/reviewers. We strongly encourage code deposition in a community repository (e.g. GitHub). See the Nature Research guidelines for submitting code & software for further information.

Data

Policy information about availability of data

All manuscripts must include a data availability statement. This statement should provide the following information, where applicable:

- Accession codes, unique identifiers, or web links for publicly available datasets
- A list of figures that have associated raw data
- A description of any restrictions on data availability

Metabolomics data have been deposited to the EMBL-EBI MetaboLights database with the identifier MTBLS1140. The complete dataset can be accessed here https://www.ebi.ac.uk/metabolights/MTBLS1140. Any further data not included in the manuscript is available from the corresponding author on reasonable request. The source data underlying Figs 2-4 and 5A-D and Supplementary Figs 2, 3C-D, 4-6 are provided as a Source Data file.

Field-specific reporting

Please select the one below that is the best fit for your research. If you are not sure, read the appropriate sections before making your selection.

- [x] Life sciences
- [ ] Behavioural & social sciences
- [ ] Ecological, evolutionary & environmental sciences

For a reference copy of the document with all sections, see nature.com/documents/nr-reporting-summary-flat.pdf

Life sciences study design

All studies must disclose on these points even when the disclosure is negative.

Sample size	We have used at least three biological replicates for all experiments. The design was based on prior assay experience and similar experiments reported in the literature.
Data exclusions	No data were excluded from the analyses.
Replication	All experiments were highly reproducible.
Randomization	Samples were processed in random order.
Blinding	All experimenters were blinded to group allocation.

Reporting for specific materials, systems and methods

We require information from authors about some types of materials, experimental systems and methods used in many studies. Here, indicate whether each material, system or method listed is relevant to your study. If you are not sure if a list item applies to your research, read the appropriate section before selecting a response.

Materials & experimental systems

n/a	Involved in the study	
☐	☒	Antibodies
☐	☒	Eukaryotic cell lines
☒	☐	Palaeontology
☐	☒	Animals and other organisms
☒	☐	Human research participants
☒	☐	Clinical data

Methods

n/a	Involved in the study	
☒	☐	ChIP-seq
☒	☐	Flow cytometry
☒	☐	MRI-based neuroimaging

Antibodies

Antibodies used	Actin antibody (1:1000 dilution, Cell Signaling Technology, catalog 4970P, lot 11); PCNA antibody (1:1000 dilution, Cell Signaling Technology, catalog 13110P, lot 2); NF-κB p65 antibody (1:1000 dilution, Cell Signaling Technology, catalog 8242S, lot 9); iNOS antibody (1:1000 dilution, Abcam, catalog ab178945, lot GR324713-6); normal rabbit IgG antibody (1:1000-1:3000 dilution, Cell Signaling Technology, catalog 7074P2, lot 27).
Validation	All antibodies had validation statements and results on the manufacturer's websites.

Eukaryotic cell lines

Policy information about cell lines

Cell line source(s)	Murine-derived macrophage RAW 264.7 cells were purchased from the Cell Bank of the Chinese Academy of Sciences (Shanghai, China).
Authentication	RAW264.7 cells are very distinct in morphology and have been tested for expression of known markers.
Mycoplasma contamination	All cells tested negative for mycoplasma contamination.
Commonly misidentified lines (See ICLAC register)	No commonly misidentified cell lines were used.

Animals and other organisms

Policy information about studies involving animals; ARRIVE guidelines recommended for reporting animal research

Laboratory animals	C57BL/6J wild-type (WT) mice were purchased from Zhejiang Vital River Laboratory Animal Technology Co., Ltd. (Jiaxing, China). Irg1-/- mice on the C57BL/6J genetic background were bought from Jackson Laboratory.
Wild animals	No wide animals were used in this study.
Field-collected samples	No field-collected samples were used in this study.
Ethics oversight	This study was approved by the Institutional Animal Care and Use Committee (IACUC) of China Pharmaceutical University Experimental Animal Center.

Note that full information on the approval of the study protocol must also be provided in the manuscript.
